# Cellular and humoral immunity in a wild mammal: Variation with age & sex and association with overwinter survival

**DOI:** 10.1002/ece3.2584

**Published:** 2016-11-15

**Authors:** Rebecca L. Watson, Tom N. McNeilly, Kathryn A. Watt, Josephine M. Pemberton, Jill G. Pilkington, Martin Waterfall, Phoebe R.T. Hopper, Daniel Cooney, Rose Zamoyska, Daniel H. Nussey

**Affiliations:** ^1^ School of Biological SciencesInstitutes of Evolutionary Biology & Immunology and Infection ResearchUniversity of EdinburghEdinburghUK; ^2^ Moredun Research InstituteMidlothianUK

**Keywords:** antibody, ecological immunology, fitness, gastrointestinal nematode, lymphocyte, *Ovis aries*, St Kilda, T cell, *Teladorsagia circumcincta*

## Abstract

Immune defenses are expected to be crucial for survival under the considerable parasite pressures experienced by wild animals. However, our understanding of the association between immunity and fitness in nature remains limited due to both the complexity of the vertebrate immune system and the often‐limited availability of immune reagents in nonmodel organisms. Here, we use methods and reagents developed by veterinary researchers for domestic ungulates on blood samples collected from a wild Soay sheep population, to evaluate an unusually broad panel of immune parameters. Our evaluation included different innate and acquired immune cell types as well as nematode parasite‐specific antibodies of different isotypes. We test how these markers correlate with one another, how they vary with age‐group and sex, and, crucially, whether they predict overwinter survival either within or among demographic groups. We found anticipated patterns of variation in markers with age, associated with immune development, and once these age trends were accounted for, correlations among our 11 immune markers were generally weak. We found that females had higher proportions of naïve T cells and gamma–delta T cells than males, independent of age, while our other markers did not differ between sexes. Only one of our 11 markers predicted overwinter survival: sheep with higher plasma levels of anti‐nematode IgG antibodies were significantly more likely to survive the subsequent high mortality winter, independent of age, sex, or weight. This supports a previous finding from this study system using a different set of samples and shows that circulating antibody levels against ecologically relevant parasites in natural systems represent an important parameter of immune function and may be under strong natural selection. Our data provide rare insights into patterns of variation among age‐ and sex groups in different T‐cell subsets and antibody levels in the wild, and suggest that certain types of immune response—notably those likely to be repeatable within individuals and linked to resistance to ecologically relevant parasites—may be most informative for research into the links between immunity and fitness under natural conditions.

## 1 Introduction

Parasites and pathogens represent a major selective force on their hosts, and the development and maintenance of immune defenses are expected to be important for survival and fitness under natural conditions (Schmid‐Hempel, 2003; Seppälä, 2015; Sheldon & Verhulst, 1996). Although positive associations between markers of immunity and survival have been observed in laboratory and domestic settings, evidence from natural populations experiencing ecologically relevant parasite challenges remains relatively scarce (Møller & Saino, 2004; Schimd‐Hempel, 2011; Seppälä, 2015; Viney, Riley, & Buchanan, 2005). Immunological studies in wild vertebrates are made particularly challenging by the staggering complexity of the vertebrate immune system, coupled with the limited range of immunological reagents available for most nonmodel species (Boughton, Joop, & Armitage, 2011; Demas, Zysling, Beechler, Muehlenbein, & French, 2011). For this reason, research to date within the field of ecological immunology has tended to rely on a relatively small number of functional immune assays that can readily be applied across species (Demas et al., 2011). However, key questions—such as how different aspects of immunity are related, and which immune parameters are most important in predicting fitness under natural conditions—can only be answered through the application of a wider range of immunological methods in the field (Boughton et al., 2011; Demas et al., 2011; Pedersen & Babayan, 2011). Here, we measure a range of immune cell types and parasite‐specific antibodies from blood samples collected from a free‐living population of mammals. We test how these immune parameters relate to one another, whether they differ among age classes and sexes, and whether they predict subsequent overwinter survival within and across these demographic groups.

The vertebrate immune system is typically regarded as consisting of the innate and adaptive arms, although interactions between these two systems are essential for an effective immune response (Murphy, Travers, & Walport, 2012). The innate immune system, which is the older in evolutionary terms, refers to nonspecific defense mechanisms which become activated immediately or within hours of antigen encounter. These defense mechanisms include anatomical barriers and behaviors to avoid infection, humoral factors such as complement and acute phase proteins, and a variety of functionally diverse white blood cells including macrophages, neutrophils, and eosinophils (Demas et al., 2011). The adaptive immune system refers to antigen‐specific immunity and is more complex than innate immunity and slower to develop. Adaptive immune responses are, however, capable of producing long‐lasting immunological protection against particular parasites as a result of immunological “memory.” The key cells are lymphocytes, composed principally of B and T cells, which are capable of discriminating host and nonhost molecular patterns and coordinating a directed immune response against parasites and pathogens (Parkin & Cohen 2001). B cells produce antibodies which play a critical role in recognizing and neutralizing infectious agents (Alberts et al. 2002). A variety of functionally distinct antibody isotypes are involved in antibody‐mediated immunity (Murphy et al., 2012). In mammals, IgG is the predominant circulatory antibody isotype, while IgA and IgE are detected at lower concentrations in circulation, but predominate at mucosal surfaces where they play an important role in immune functions (Manz, Hauser, Hiepe, & Radbruch, 2004). Most mature alpha–beta (αβ) T cells are produced in the thymus, emerging in a “naïve” state and activating to become effector or memory T cells when they recognize a peptide expressed in conjunction with an MHC molecule at the surface of a host cell and receive appropriate stimulatory signals from other immune cells (Murphy et al., 2012). Gamma–delta T cells, which are rare in the circulation in humans but more common in ruminants and poultry, emerge from the thymus in an effector state, are not restricted to recognizing antigen presented with MHC, and can produce rapid, localized responses (Bonneville, O'Brien, & Born, 2010). A wide range of αβ T‐cell subtypes with distinct functions have been described based on specific expression of intra‐ and extracellular markers and quantitatively evaluated in using techniques, such as flow cytometry (Murphy et al., 2012). Very broadly, these subtypes include cytotoxic T cells which recognize and destroy cells with intracellular pathogens, helper T cells which coordinate immune response to infection, and regulatory T cells (Treg) which play a key role in maintaining peripheral immunological tolerance by actively suppressing the immune response (Muehlenbein, 2010). To our knowledge, no study of a wild vertebrate has simultaneously measured variation in different T‐cell subtypes, antibody levels, and innate immune cell numbers and related these different aspects of the immune phenotype to survival.

Profound changes in immune phenotype and function are observed over the course of an organism's lifespan, with resistance to infection typically developing through early life into adulthood and certain aspects of immune function becoming compromised in old age (Simon, Hollander, & McMichael, 2015). Variation in markers associated with immune function with age has been widely reported in wild vertebrates (Cichoń, Sendecka, & Gustafsson, 2003; Jego et al., 2014; Nussey, Watt, Pilkington, Zamoyska, & McNeilly, 2012; Palacios, Winkler, Klasing, Hasselquist, & Vleck, 2011). Notably, the structure and function of the thymus in mammals and in birds deteriorates remarkably early in life in many vertebrates and the output of naïve T cells may be greatly reduced by the time an individual reaches sexual maturity (Cockburn, 1992; Møller & Erritzøe, 2001). Although age‐dependent declines in circulating naïve T cells have been widely observed in laboratory and domestic mammals and humans, the fitness consequences of variation in the availability of naïve T cells at different ages under natural conditions are completely unknown (Shanley, Aw, Manley, & Palmer, 2009). Sex differences in immune function are also predicted by evolutionary theory and there is some evidence to support these predictions from natural and laboratory systems (Zuk & McKean, 1996). A major challenge for our understanding of how variation in immunity impacts evolutionary and population dynamics of natural systems, given the expectation and observation of age and sex differences in immunity, is therefore to test whether the associations between measures of immunity and fitness differ among demographic groups.

In this study, we measured differential white blood cell counts, used ELISA to assay parasite‐specific antibody levels, and used flow cytometry to differentiate T‐cell subtypes to generate a broad panel of 11 immune markers in a cross‐sectional sample spanning all ages and both sexes in a population of free‐living Soay sheep (*Ovis aries*). Our work builds on a previous study which documented patterns of age‐related change in T‐cell subtypes consistent with those observed in the human, laboratory rodent, and domestic ruminant literature, but had insufficient sample sizes to meaningfully test for associations with fitness measures independently of age (Nussey et al., 2012). We have also previously shown that antibodies against a highly prevalent gastrointestinal nematode parasite in our study population, *Teladorsagia circumcincta* (Tc), are negatively associated with parasite egg counts and positively associated with overwinter survival in adult Soay sheep (Coltman, Wilson, Pilkington, Stear, & Pemberton, 2001; Hayward et al., 2014; Nussey et al., 2014). Here, we test how our immune markers, which encompass a range of different innate and immune cell types, are correlated with one another; how they vary with age and sex; and to what extent the markers predict overwinter survival and whether associations with survival are age or sex dependent.

## 2 Materials and Methods

### 2.1 Study system & field data collection

Soay sheep are a primitive breed of domestic sheep, which has dwelt unpredated and unmanaged in the remote St Kilda archipelago for several millennia. The animals resident to the Village Bay area of the main island of Hirta within the archipelago have been the focus of a long‐term individual‐based study since 1985 (Clutton‐Brock & Pemberton, 2004). These individuals are caught and marked at birth, and their life histories are closely monitored from birth to death. Most are caught once a year during summer for sampling and measurement. The population exhibits a distinctive, unstable dynamic characterized by low and rising sheep numbers followed by high mortality (“crash”) winters in which more than half of the population may perish (Clutton‐Brock & Pemberton, 2004). High mortality winters are associated with strong selection on a range of phenotypic traits and are thought to result from a combination of low food availability due to competition, harsh winter climate conditions, and parasite pressure, predominantly from Strongyle gastrointestinal nematodes (GINs) (Coulson, 2001; Gulland, 1992; Gulland & Fox, 1992). Age‐related variation is well understood in this population, with differences evident between lambs, yearlings, prime age adults (2–6 years), and geriatrics (>6 years) in demographic rates and phenotypic traits both within and among the sexes (Coulson, 2001).

Samples and data for this study were collected in August 2011, during the Soay sheep research project's annual summer catch. During a 2‐week period, 287 marked individual sheep were rounded up, caught and processed in a series of corral traps set up in the Village Bay area. Our sample comprised 50 male and 49 female lambs (approximately 4 months old), 5 male and 18 female yearlings (1 year and 4 months), 40 male and 93 female adults (2–6 years), and 4 male and 28 female geriatrics (seven or more years). Upon capture, each individual was weighed and measured and then blood and fecal samples were collected. Fecal samples were used to measure Strongyle and Strongyloides parasite fecal egg counts (FEC), using a modification of the McMaster technique (Gulland & Fox, 1992; Ministry of Agriculture Fisheries and Food 1971). Two 9‐ml Li‐heparin Vacutainer tubes of whole blood were taken from each individual and stored at 4°C until processing. One Vacutainer was used for plasma extraction for antibody analysis, and the other was used for differential cell counts and leukocyte fixation for subsequent staining for flow cytometry. In the case of the single γδ+ stain, this was 2 months, and for the single CD4+ & CD8+, the triple (CD4+ naïve and CD8+ naïve), and intracellular (Foxp3+ Treg) stains, this was 7 months. Despite the variation in time for some stains between collection and analysis by flow cytometry, we confirmed that cell populations were correlated between the two time points (*r* = .56 for CD4+ and *r* = .71 for CD8+). The winter of 2011/2012 was a high mortality “crash” winter on Hirta. Winter censuses and mortality searchers in the study area allowed us to ascertain which of the animal's sampled in August 2011 died over the following winter. A total of 139 of our 287 sampled individuals (48%) had died of natural causes by 1 May 2012.

### 2.2 Laboratory methods

#### 2.2.1 Differential white blood cell counts

Within 12 hr of collection, 5 μl of whole blood was applied on to one end of a standard glass microscope slide. The drop of blood was then spread at a 45° angle and drawn across the slide to produce an even film. Slides were air‐dried overnight and stained using a Quick‐Diff Kit stain (Gentaur) the following day as per manufacturer's instructions. A total of 100 cells were counted at 40× magnification using the battlement track method and based on staining and morphology, identified as either lymphocytes, eosinophils, or neutrophils (Bain, 2006). Basophils and monocytes were observed too rarely to analyze. Only slides with a clear regular monolayer of cells were counted. Slides with uneven cell density or unclear staining were omitted from analysis (20%, see Table 1). From our counts, we calculated a total eosinophil count and the neutrophil to lymphocyte ratio (NLR).

**Table 1:** Results of linear models testing age and sex effects on each of the 11 immune parameters measured in this study. Global F tests of the significance of the age‐by‐sex interaction, and the age‐group and sex main effects are reported. Interactions between sex and age‐group were tested but were not significant in any case, so main effects of sex and age‐group are reported with the interaction dropped from the model. Estimated differences in the mean among age‐ and sex groups are reported along with standard errors of differences in brackets, expressed as difference from the female lamb group. The R‐squared value refers to the model with age and sex included as main effects only

Immune marker	Global tests			Post hoc comparisons					*r* ^2^
	Age x Sex	Age	Sex	Intercept (female lambs)	vs. males	vs. yearlings	vs. adults	vs. geriatrics	
Neutrophil: Lymphocyte	2.213	8.343***	1.159	0.798 (0.126)	0.147 (0.137)	0.140 (0.228)	0.613 (0.144)	0.819 (0.217)	.087
Eosinophil	0.461	17.346***	1.324	2.085 (0.560)	−0.699 (0.608)	2.392 (1.015)	4.512 (0.642)	4.111 (0.968)	.201
CD4+	0.854	7.424***	0.088	25.185 (1.464)	−0.489 (1.647)	6.539 (2.563)	4.287 (1.671)	11.510 (2.535)	.097
CD8+	0.117	7.806***	0.217	5.617 (0.464)	−0.246 (0.528)	−0.918 (0.811)	1.593 (0.535)	2.987 (0.882)	.103
CD4+ Naive	0.440	72.022***	10.707**	47.392 (1.606)	−5.936 (1.814)	−20.426 (2.808)	−25.229 (1.847)	−30.845 (2.988)	.544
CD8+ Naive	0.525	32.524***	4.462*	73.020 (2.296)	−5.480 (2.594)	−10.953 (4.016)	−23.673 (2.641)	−35.205 (4.274)	.373
*γδ*+ TcR	0.401	112.87***	12.42***	26.796 (0.856)	−3.353 (0.951)	−7.928 (1.450)	−16.404 (0.970)	−19.180 (1.435)	.655
Treg	0.742	1.221	0.004	4.0504 (0.588)	−0.042 (0.662)	−1.410 (1.013)	−1.170 (0.672)	−0.532 (1.035)	−.002
Anti‐Tc IgA	0.763	28.970***	0.508	0.168 (0.031)	−0.024 (0.034)	0.215 (0.060)	0.286 (0.035)	0.404 (0.054)	.248
Anti‐Tc IgE	2.311	44.195***	0.178	0.041 (0.012)	−0.006 (0.013)	0.085 (0.024)	0.127 (0.014)	0.221 (0022)	.332
Anti‐Tc IgG	0.664	82.609***	0.010	0.325 (0.032)	−0.003 (0.035)	0.625 (0.062)	0.537 (0.036)	0.452 (0.056)	.480

#### 2.2.2 Antibody measures

Within 24 hr of collection, one Vacutainer of whole blood was centrifuged at 1008 g for 10 min and the plasma layer removed and stored at −20°C. We then followed previously published methods to measure levels of IgE, IgA, and IgG antibodies binding larval stage 3 antigens from *T. circumcincta*, a highly prevalent gastrointestinal Strongyle nematode parasite of Soay sheep on Hirta (Nussey et al., 2014), full details of which are provided in the Supplementary Methods file.

#### 2.2.3 T‐cell subsets

Lymphocytes were preserved in fixative after collection on St Kilda and later analyzed, following antibody labeling, by flow cytometry in Edinburgh (following Nussey et al., 2012). Briefly, red cells were removed from 1 ml of whole blood by addition of 5 ml of ammonium chloride lysing solution (1.5 mol/L NH4Cl, 100 mmol/L NAHCO3, 10 mmol/L Na2EDTA), which was added to 1 ml of whole blood and mixed gently before centrifuging at 1008 g for 10 min. The supernatant was removed and the cell pellet resuspended in 9 ml phosphate‐buffered saline (PBS) to wash. The sample was then spun, the supernatant removed, and the pellet resuspended in 2.5 ml 1% paraformaldehyde (PFA) in PBS at room temperature for 10 min. After the cells were spun at 1008 g for 10 min, the supernatant was removed and the cell pellet resuspended in 9 ml phosphate‐buffered saline (PBS) to wash. The cells were then spun again at 1008 g for 10 min and finally resuspended in PBS + 0.02% NaN3 solution. The samples were stored at 4°C until staining and analysis by flow cytometry. This is a lengthy process and time constraints during our intensive August field season on St Kilda meant that only 200 out of 287 available samples could be processed.

Flow cytometry protocols broadly followed those described in Nussey et al. (2012) with some modifications as described in full in the Supplementary Methods file. Fluorescently labeled monoclonal antibodies were used to identify the proportions of T helper cells (CD4+), cytotoxic T cells (CD8+), and gamma–delta T cells (γδ TcR+) within the total lymphocyte population within each sample and the proportion of T helper cells which were Treg (FoxP3+). Naïve helper and cytotoxic T cells were identified by their co‐expression of the CD45RA marker, and the subpopulation of regulatory T helper cells which were Treg (FoxP3+) was identified by co‐expression of CD4+ and FoxP3+ antibodies. Flow cytometry data were analyzed using FlowJo version X.0.7 analysis software (TreeStar, San Carlos, CA, USA). Proportions of T cells were measured by firstly placing a gate encompassing the entire lymphocyte population. T‐cell populations were then gated using specific CD4, CD8, or gamma–delta fluorescence, followed by appropriate subtype gating based on CD45RA and FoxP3 fluorescence. An example of our gating strategy is presented in Figures S1. To ensure robust quality control, only samples with sufficient numbers of positive cells (>100) were included in analysis and those with poor staining, poor cell profiles or low cell numbers were omitted from analysis (see Table S1 for data available for each T‐cell subset).

### 2.3 Data analysis

Available sample sizes varied among immune assays for various reasons, discussed above. A small number of extreme outliers were identified in the raw data, and because these lay outside what seemed a biologically plausible range based on previous studies (Egbe‐Nwiyi, Nwaosu, & Salami, 2000; Holman, 1944; Nussey et al., 2012; Pisek, Travnicek, Salat, Kroupova, & Soch, 2008), we excluded them from further analyses. This included 2 data points for NLR (both >6), 2 for eosinophil counts (>30%), 2 for CD8+ (>25% of lymphocytes), and one for CD4+FoxP3+ (>65% of CD4+ T cells). The final available sample sizes for each marker are presented in Table S1.

The immune measures generally approximated a normal distribution and, in the few cases where skew was evident, our results were unchanged when we used log_10_ transformations and so we therefore present results from untransformed data. All analyses were conducted in R version 3.1.3 (R Core Team 2015). We estimated the Pearson's correlation coefficient for all pairs of immune markers, as well as exploring the dimensionality of the data using principal components analysis (PCA). Our further analyses, as well as previous studies (Nussey et al., 2012), suggested many of the markers differ among age‐groups and our PCA suggested a main axis of variation associated with age (see Results). Following previous demographic and immunological work on this population, we elected to consider discrete age‐groups of animals in our analyses (Coulson, 2001; Nussey et al., 2012). We examined variation among lambs, yearlings, adults (2–6 years), and geriatrics (>6 years). To capture covariation among markers within age‐groups, we reran our correlation analyses using residuals from a model of each marker including age‐group as a factor. We went on to test how each marker varied with age‐group and sex using separate linear models (LMs) including an age‐group‐by‐sex interaction. This interaction was removed from the model if found to be nonsignificant based on a likelihood ratio test, and the significance of the main effects of age‐group and sex were then tested.

We tested whether our immune measures predicted overwinter survival by fitting generalized linear models (GLMs) of survival as a binary variable (coded one for survivors, zero for animals that died) with a binomial error distribution and a logit link function. As previous studies have suggested that overwinter survival varies with age, sex, August weight, and FEC, we initially examined the effects of these terms in our sample (Coulson, 2001; Gulland, 1992). We found evidence for a significant age‐by‐sex interaction (χ^2^ = 9.84, *P* = .02) with highest survival in female and adult groups, as expected. We also found a significant positive association with August weight (χ^2^ = 10.98, *P* = .001), but did not find a significant association with August FEC (χ^2^ = 2.746, *P* = .098) independent of weight. We therefore tested for associations among our immune markers and survival by adding them to a GLM including an age‐group‐by‐sex interaction and August weight, either separately, in groups (differential cell count data; FACS data; antibody data) or all together. When more than one immune variable was added to the model, the variable with the lowest likelihood ratio test (LRT) statistic upon removal from the model was sequentially deleted from the model until only terms significant at *P* < .05 remained. Each removed variables were then added back into the final model sequentially and the LRT was used to confirm that the markers omitted did not significantly increase the explanatory power of the model.

## 3 Results

**Figure 1:**
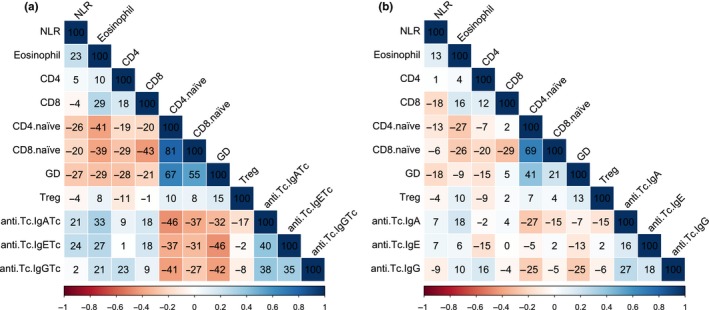
Correlation matrices showing pairwise Pearson's correlation coefficients among all 11 immune parameters. (a): Raw data and (b): correlations among residuals from a model of the immune parameter including age‐group as a factor. The strength of each pairwise correlation coefficient is represented in the strength of the color, which is blue for positive correlations and red for negative correlations. For ease of visualization, the numbers in the boxes have been *100. Abbreviations refer to neutrophil: lymphocyte ratio (NLR) and gamma–delta T cells (GD)

**Figure 2:**
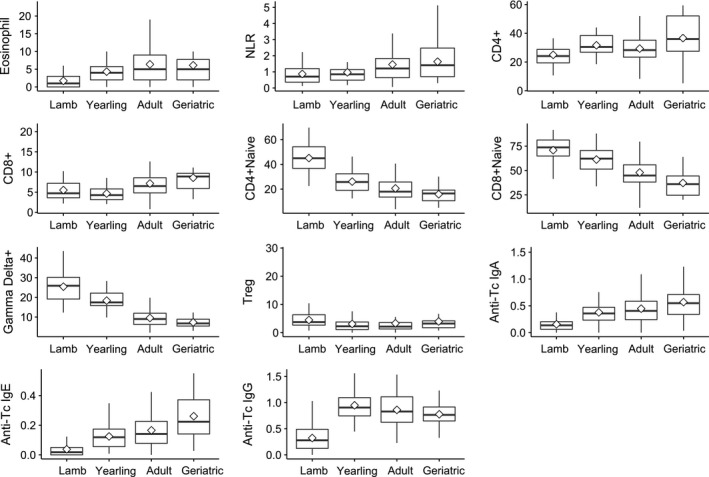
Variation in immune parameters among age‐groups. The mean for each age‐group is represented by a diamond shape within each box and the median by a black horizontal line. The box represents the interquartile range (IQR) and the whiskers show the highest and lowest values within 1.5*IQR. Measures included are as follows: eosinophil count (“Eosinophil”), neutrophil: lymphocyte ratio (NLR), proportion of lymphocytes which are “CD4+” and “CD8+”, and the proportion of these subsets which are, in turn, CD45RA+ (“Naïve”) or FoxP3+ (“Treg”), the proportion of “gamma–delta+” T cells, and the optical density scores for antibodies against *Teladorsagia circumcincta* (“Anti‐Tc”) of isotypes IgG, IgA, and IgE

There was evidence for some moderate correlations (*r* > .4) among the 11 immune markers, but these were predominantly driven by age‐dependent variation in those markers (Figure 1). The strongest positive associations were found among the two naïve T‐cell types (CD4+ and CD8+) and gamma–delta T cells on the one hand, and eosinophil counts and the three anti‐Tc antibody measures on the other, with negative correlations present between these two groups of markers (Figure 1a). The first axis of our PCA explained 35% of the overall variation, but subsequent axes all explained only 12% or less (Table S2). The first PCA axis had heavy positive loadings from gamma–delta T cells, the two naïve T‐cell types, and negative loadings from eosinophil counts and the Tc antibody measures (Table S2). We found that the markers with positive loadings on PC1 declined strongly with age, while the markers with negative loadings increased with age (Figure 2 and below), suggesting most of the observed correlation structure among the measures was associated with age. Supporting this, correlations among immune measures corrected for age differences (using residuals from models including age‐group as a factor) were considerably reduced, although associations among the naïve T cells and gamma–delta cells remained moderately high and positive within age‐groups (*r* > .4; Figure 1b).

**Figure 3:**
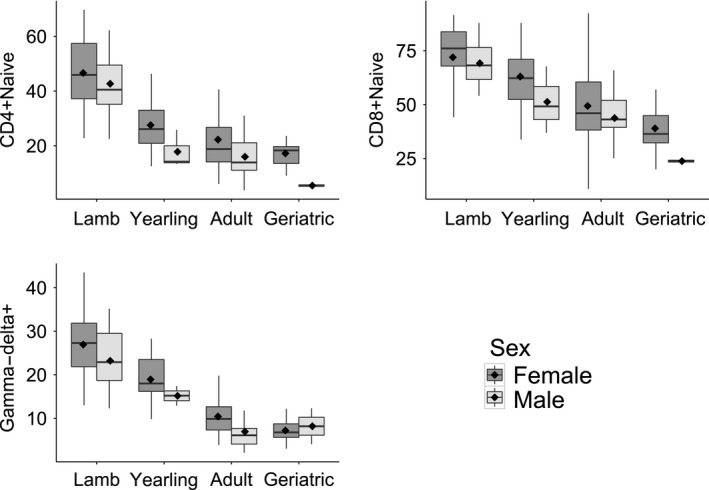
Sex differences observed in three of the 11 immune parameters: naïve T cytotoxic (CD8+), naïve T helper (CD4+), and gamma–delta T cells. In all three parameters shown, females have higher proportions of each cell type than males, and this difference was found to be independent of age (see Table 3). The mean for each age‐group is represented by a diamond shape within each box and the median by a black horizontal line. The box represents the interquartile range (IQR) and the whiskers show the highest and lowest values within 1.5*IQR

All immune parameters except regulatory T cells varied significantly with age‐group, and three (naïve T helper, naive T cytotoxic, and gamma–delta T cells) differed between the sexes (Figures 2 and 3, Table 1). We found no evidence for age‐by‐sex interactions in any of the immune parameters (Table 1). Eosinophil counts and anti‐Tc antibodies increased progressively from the lamb to adult age‐groups, while NLR and the proportion of CD8+ (cytotoxic) and CD4+ (helper) T cells were higher in adults and geriatrics than in lambs (Table 1, Figure 2). The proportion of naïve T cells in both helper and cytotoxic T‐cell subsets and the proportion of gamma–delta T cells showed a progressive decline across the age‐groups and were also higher in females than males (Figures 2 and 3, Table 1).

**Figure 4:**
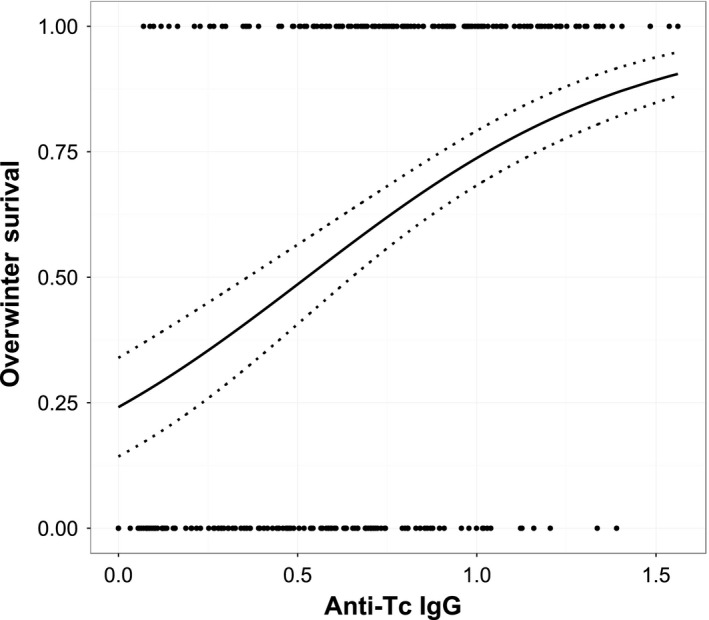
A logistic regression plot of the relationship between August anti‐*Teladorsagia circumcincta* IgG antibody levels and subsequent overwinter survival (1 = survived, 0 = died). The points in the plots are the raw data and include all age‐groups and sexes combined. The black line is of the predicted values for adult females from the final model. The dotted lines show the standard error of these values

The only immune measure which significantly predicted subsequent overwinter survival was anti‐Tc IgG levels. Individuals with higher levels of these antibodies were more likely to survive, independent of age‐group, sex, and weight (Table 2, Figure 4). There was no evidence of sex‐by‐immune measure interactions and none of the other immune parameters were significant when fitted alone in the survival GLMs (Table 2). This result was unaffected when we included multiple immune parameters as either groups or altogether and simplified the GLMs: The only measure remaining in our simplified model was anti‐Tc IgG antibody level.

**Table 2:** Generalized linear models of overwinter survival including an age‐by‐sex interaction and August weight (see Results section for details) with each immune parameter separated included and tested. A sex‐by‐immune parameter interaction was tested and dropped where nonsignificant, and the slope (with standard error in brackets) and likelihood ratio test statistic is reported for each immune parameter fitted separately

Immune marker	Sex*Immune marker	Immune marker	
	*χ* ^2^	b (*SE*)	χ^2^
Neutrophil: Lymphocyte	0.244	−0.291 (0.172)	2.854
Eosinophil	0.319	−0.036 (0.037)	0.956
CD4+	1.775	−0.013 (0.018)	0.565
CD8+	0.927	−0.007 (0.055)	0.017
CD4+ naïve	0.690	0.015 (0.017)	0.848
CD8+ naïve	3.603	0.018 (0.012)	2.288
*γδ*+ TcR	0.303	0.052 (0.035)	2.275
Treg	0.172	0.030 (0.044)	0.488
Anti‐Tc IgA	0.494	0.648 (0.550)	1.428
Anti‐Tc IgE	0.002	0.723 (1.354)	0.287
Anti‐Tc IgG	1.844	2.179 (0.577)	15.505***

## 4 Discussion

We have measured an unusually broad range of immune cell types and antibody isotypes in a wild vertebrate population, facilitated by the availability of reagents from veterinary immunology for our study species. Once the strong and expected age‐related changes in our immune measures had been accounted for, correlations among our 11 markers were weak. Other vertebrate studies measuring many immune markers have reported generally weak correlations and complex patterns of association between these measures and resistance or health outcomes (Banos et al., 2013; Buehler, Versteegh, Matson, & Tieleman, 2011; Flori et al., 2011; Keil, Luebke, & Pruett, 2001; Matson, Cohen, Klasing, Ricklefs, & Scheuerlein, 2006). This suggests that one or a few measures of immunity are unlikely to capture broadscale variation in immune responsiveness and the ability to resist parasites, and raises the important question of which of the potentially huge range of immune markers are likely to be most relevant for ecological and evolutionary studies (Boughton et al., 2011; Demas et al., 2011). We found that the only immune marker to predict survival over the subsequent winter on St Kilda was the abundance of IgG antibodies against *T. circumcincta*. The strong, positive association of this marker with survival was independent of age, sex, body mass, and FEC, and affirms an identical result from a study of adult female Soays sampled across three previous crash years (Nussey et al., 2014). Previous work in this system has demonstrated a negative linear relationship between anti‐GIN antibody measures and FEC, and this combined with the confirmation of a positive association with survival supports the hypothesis that individuals with high anti‐Tc IgG antibodies are more resistant to nematode infection (Hayward et al., 2014; Nussey et al., 2014). Below, we discuss the implications of the observed age and sex trends in our immune measures and offer potential reasons why this particular marker, and not others, was found to be predictive of survival.

The strong increases in antibody measures and declines in naïve T‐cell and gamma–delta T‐cell subsets with age observed are all consistent with patterns of normal immunological development. The development of immunity to gastrointestinal nematodes (GIN) parasites including Tc is thought to be antibody‐mediated in sheep (Stear, Park, & Bishop, 1996), and experimental studies show that antibody responses increase with age as the immune response develops (Nguyen, 1984; Smith, Jackson, Jackson, & Williams, 1985; Watson, Colditz, Andrew, Gill, & Altmann, 1994). All individuals are exposed to these parasites from very early life on St Kilda (Wilson, Grenfell, Pilkington, Boyd, & Gulland, 2004), so it is likely that the population‐level increase in antibody levels reflects the steady development of the immune response to these worms over the animals’ first few years. Clear and progressive declines in the proportions of naïve T cells have previously been reported in this population from a smaller sample of animals (Nussey et al., 2012). We expected and observed that circulating proportions of naïve T cells declined with age as the supply of naïve cells reduces with thymic involution and as the pool of naïve cells activate to become mature effector or memory T cells over time (Aspinall & Andrew, 2000; Cunningham, Kimpton, Holder, & Cahill, 2001). In young domestic ruminants, the proportions of circulating gamma–delta T cells are relatively high, up to an order of magnitude greater than those seen in mice and humans, but decline sharply with age (Hein & Mackay, 1991), a pattern also previously observed in Soay sheep (Nussey et al., 2012). This is presumably the result of a drop in thymic output of these cells related to thymic involution and their continuous removal from the circulatory system over time. Although within‐individual immune development seems the most likely explanation for these patterns, our data are cross‐sectional and so we cannot exclude the role of among‐individual processes, such as annual variation in exposure to parasites or selective appearance or disappearance.

This is the first study to report sex differences in naïve and gamma–delta T‐cell subpopulations in a wild mammal. We found that females had higher proportions of these T‐cell subtypes than males, independent of age. Similar patterns have been observed in laboratory rodents and in humans (Caccamo, Dieli, Wesch, Jomaa, & Eberl, 2006; Pido‐Lopez, Imami, & Aspinall, 2001; Scotland, Stables, Madalli, Watson, & Gilroy, 2011). Human females have higher circulating levels of Vγ6/Vδ2+ T cells, which are the major component of human peripheral gamma–delta T cells (Caccamo et al., 2006). When aged between 20 and 62 years, women also have a higher thymic output than age‐matched men, although the absolute number of T cells did not differ between the sexes (Pido‐Lopez et al., 2001). Sex hormones have been shown to affect the rate and maintenance of thymic output in humans (Ansar Ahmed, Penhale, & Talal, 1985; Dumont‐Lagace, St‐Pierre, & Perreault, 2015), resulting in higher thymic output in adult females than males (Gui, Mustachio, Su, & Craig, 2012), and could be behind the sex difference in naïve and gamma–delta T cells in Soay sheep. It is interesting to note that, as in humans and many other polygynous mammals, Soay sheep females have lower annual mortality and longer lifespan than males (Clutton‐Brock & Pemberton, 2004). Conceivably, reduced thymic output in males could reflect weaker selection to maintain certain aspects of immune function into later adulthood compared to longer‐lived females. Whether there is any fitness cost of reduced thymic output and whether the cost differs between the sexes remain interesting questions for further study, although in our cross‐sectional data, there was no association during adulthood between naïve or gamma–delta T‐cell proportions and survival in either sex.

Given the prediction that robust immune defenses are essential for survival in parasite‐rich natural environments, why did only one of our eleven immune parameters significantly predicted overwinter survival? What do levels of circulating anti‐Tc IgG antibodies tell us about host immunity in this population that our other immune parameters do not? In our study population, gastrointestinal nematodes appear to represent the major parasite pressure (Craig, Pilkington, & Pemberton, 2006; Graham et al., 2016). Anti‐Tc antibodies measure levels of circulating immunoglobulins with known effector function against a specific ecologically important parasite, as opposed to our other measures which characterize the relative proportions of functionally distinct immune cell types. These latter measures do not differentiate which, if any, parasites these different cells are reacting to or fighting, nor how well they are functioning in that capacity. Furthermore, infection is associated with the sequestration of immune cells from circulation to sites of infection. While nematode‐specific antibodies in blood correlate with mucosal antibody activity at the site of infection in sheep (Prada Jiménez de Cisneros, Matthews, Mair, Stefan, & Stear, 2014), it is not clear whether the proportions of different leukocytes measured here reflect only the available immune cell selection pool rather than the mucosal effector population. The well‐established complexity of the vertebrate immune system, which is clearly illustrated by the weak age‐independent correlations among our 11 immune markers, represents a major challenge for eco‐immunologists seeking to identify a handful of salient immune measurements to study in the field (Boughton, Joop & Armitage 2011; Demas et al., 2011). Our results suggest that antibodies specific to prevalent parasites or pathogens may offer the most insight in terms of their relationship with health and fitness.

We previously found that plasma levels of anti‐Tc IgG, but not IgA or IgE, predicted overwinter survival in adult female Soays (Nussey et al., 2014), and the present study confirms this finding, showing it to be age and sex independent. Despite IgA and IgE isotypes having well‐documented roles in the development of resistance to GIN parasites in young domestic sheep (Schallig, 2000), they have short half‐lives in circulation relative to IgG and may be present in plasma at high levels only during acute infections (Manz et al., 2004). IgG may therefore represent a more temporally stable measure of anti‐GIN immunity in blood than other isotypes, a suggestion supported by the presence of significant repeatability within individuals across years of a pan‐isotype anti‐Tc antibody measure, which would have predominantly measured IgG due to its abundance in plasma (Hayward et al., 2014). These findings add weight to our previous suggestion that anti‐Tc IgG levels in summer may provide a repeatable indicator of an individual's ability to cope with established GIN infections, and therefore predict their ability to survive the interacting nutritional, thermoregulatory, and parasitological challenges of a “crash” winter on St Kilda (Nussey et al., 2014). Overall, our data suggest that immune measures which capture among‐individual variation in responses to ecologically relevant parasites and are repeatable within individuals over time may offer the clearest insights into the relationship between immunity and fitness under natural conditions.

Our findings provide novel insight into patterns of variation across immune cell types in a wild vertebrate. Although we could find no association between any T‐cell subpopulations and overwinter survival, ours is a relatively small and cross‐sectional data set and much further work is required to determine whether and how measures of T‐cell phenotype and function relate to fitness under natural conditions. It may be that the T‐cell phenotypes used in this study were too broad or inappropriate to detect ecologically important relationships. For example, it may be that total numbers of each T‐cell subset or their antigen specificity are more relevant to survival and fitness than the proportion of the T‐cell subsets within the peripheral lymphocyte pool. Studies of T‐cell function in response to ecologically relevant stimulus ex vivo have been conducted in domestic ruminants (McNeilly, Devaney, & Matthews, 2009) and wild mammals (Beirne, Waring, Mcdonald, Delahay, & Young, 2016), and could be an important indicator of immune function in natural systems. It is also worth noting that survival is only one aspect of lifetime fitness and our measures were taken several months before the onset of winter when the animals actually die. Although a longitudinal study in cattle suggests that many of the T‐cell measures we identified are highly repeatable within individuals (Banos et al., 2013), this remains to be established under natural conditions. Our data are cross‐sectional and it may be that longitudinal changes in some of these parameters (i.e., year‐on‐year decline within the lifetime of an individual), rather than measures at a single point in time, are most informative in terms of the individual's immune health and fitness. Longitudinal studies incorporating a wide range of immune parameters and linking them to health or fitness remain rare (although see Keil et al., 2001; Banos et al., 2013), but are very important for developing our understanding of how immunity and fitness relate to one another over entire lifetimes.

## Data Accessibility

Data available from the Dryad Digital Repository: doi: 10.5061/dryad.9ct33.

## Conflict of Interest

None declared.
