# Supplementary figures and images for: Cellular and humoral immunity in a wild mammal: Variation with age & sex and association with overwinter survival
Source: Ecol Evol. 2016 Nov 15;6(24):8695–705. doi: 10.1002/ece3.2584 (PMC5192870; doi:10.1002/ece3.2584)

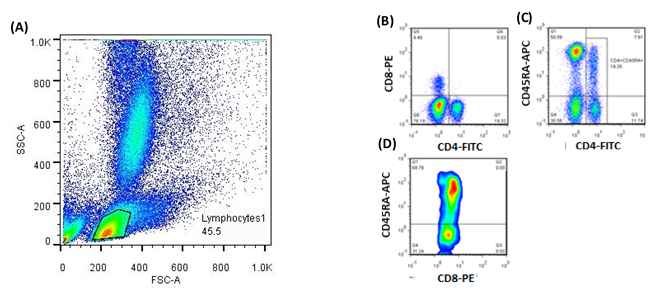

Supplement: Supplementary file 1 [file ECE3-6-8695-s001.png]
