# Supplementary material for: Cellular and humoral immunity in a wild mammal: Variation with age & sex and association with overwinter survival
Source: Ecol Evol. 2016 Nov 15;6(24):8695–705. doi: 10.1002/ece3.2584 (PMC5192870; doi:10.1002/ece3.2584)
Supplement: Supplementary file 2 [file ECE3-6-8695-s002.docx]

**Supplementary Methods**

Anti-*T.circumcintca* Antibodies ELISA methods: Samples were analysed using the same protocol and procedure as in Nussey *et al.* (2014) with minor adaptations. We used Tc L3 somatic antigen, diluted to 2µg per ml of 0.06M Carbonate buffer at pH 9.6. L3 somatic antigen was prepared by re-suspending Tc L3 in PBS (~5 x 10^5^ larvae per ml) in Lysing Matrix D tubes (MP Biomedicals) and homogenising in a Precellys^®^ 24 tissue homogeniser. Debris was pelleted by centrifugation at 16, 000 × *g* at 4^o^C and the somatic antigen containing supernatant stored at -80^o^C prior to use. Total protein concentration of the L3 antigen preparation was estimated using a Pierce™ BCA Protein Assay Kit (Thermo Scientific).

In each assay, 50µl of appropriately diluted antigen solution was added to each well of a Nunc immuno 96-microwell plate, which was subsequently covered and incubated overnight at 4^o^C. The wells were then washed three times in Tris-buffered saline-Tween (TBST) using a plate washer. Then 50µl of an appropriately diluted Soay sheep plasma sample was added to each well. Sample dilutions used (adapted from optimisation procedure described by (Nussey *et al.* 2014)) were as follows: anti- Tc IgA: 1:50; anti- Tc IgG: 1:12800; anti -Tc IgE: 1:50.

The plates were then covered and incubated at 37^o^C for 1 hour and then washed five times with TBST. For the anti-Tc IgA & anti-Tc IgG assays 50µl per well of the appropriate rabbit anti-sheep detection antibody conjugated to horseradish peroxidise (HRP) was added (anti-ovine IgA-HRP diluted 1 µl in 8mls in TBST, anti-ovine IgG-HRP diluted 0.5 µl in 8mls in TBST: all AbD Serotec, cataologue numbers: AHP949P and 5184-2504, respectively). For the anti-Tc IgE assay50µl of anti-ovine IgE (mouse monoclonal IgG1, clone 2F1, [2]) diluted 1:100 in TBST was added to each well, followed by 1 hour incubation at 37^o^C, five washes with TBST and then the addition of 50µl of goat anti-mouse IgG1-HRP detection antibody (AbD Serotec catalogue number: STAR132P), diluted to1µg in 8000ul of TBST to each well. All plates were then covered and incubated at 37^o^C for 1 hour. They were then washed five times with TBST and 100µl of SureBlue TMB 1-Component microwell peroxidase substrate (KPL) was added per well and then left to incubate for 5 minutes in the dark, in a cardboard box, at 37^o^C. Reactions were then stopped by adding 100µl 1M HCl and optical densities (ODs) were read immediately at 450nm using a Thermo Scientific Multiskan GO Spectrophotometer.

Each assay on each selected Soay sheep plasma sample was performed twice on separate ELISA plates. On each plate we also included four sample-free wells as a duplicate negative control (TBST: 200ml of 10xTrisBuffered Saline in 1800mls distilled water with 1ml Tween 20) and a duplicate positive control (Moredun Research Institute (MRI) positive sample: purified lymph from Tc infected sheep). We excluded samples across all assays for which there was obviously poor correspondence across duplicate OD scores, presumably due to human error (n=2 Anti-Tc IgA, n=1 Anti-Tc IgG). We then checked the correlation of ODs across duplicate plates and re-ran both plates if r < 0.80. For subsequent analyses, we took the average OD across the duplicate runs minus the average of the two negative control well ODs across the two plates as our assay measure.

Flow cytometry methods: Using a 96-well plate 100ul of each sample of fixed white blood cells was spun at 2000rpm for 1 minute at 4°C and the supernatant discarded. The cells were re-suspended in 200ul of 20% NGS and the plate was incubated at 4°C for 30 minutes before spinning at 2000rpm for 1 minute.

For the single colour stains cells were re-suspended and incubated at 4°C for 30 minutes with 100ul of anti-ovine monoclonal antibodies (mAb) to either γδ TcR (clone 86D, mouse IgG1, (Mackay, Beya & Matzinger 1989)), CD4 (clone 17D, mouse IgG1, (Mackay *et al.* 1988)) or CD8 (clone 7C2, mouse IgG2a, (Young *et al.* 1997)) or the appropriate isotype control antibody (mouse IgG2a and mouse IgG1 k ). The plate was washed by adding 100ul FACS buffer, spinning at 2000rpm for 1 minute, adding 200ul FACS buffer and a final spin at 2000rpm for 1 minute. Cells were then re-suspended in 100ul of secondary antibody (goat anti-mouse IgG-Alexa 647 [H+L], Invitrogen, Carlsbad, CA) and the plate incubated for 30 minutes at 4°C. The plate was then washed as described previously and the cells suspended in 200ul PBS solutions before spinning at 2000rpm for 1 minute. Cells were fixed by adding 200ul 1% PFA in PBS and incubating the plate at room temperature for 10 minutes before spinning and re-suspension in 200ul PBS solution. At this point the plate is covered and stored at 4°C until being read on a BD FACSCanto II® flowcytometer (BD Biosciences, San Jose, California, USA) within 48 hours.

For the triple colour stains cells were re-suspended and incubated at 4°C for 30 minutes with 100ul of anti-ovine mAb to either CD8 (clone 7C2, mouse IgG2a, (Young *et al.* 1997)), CD45RA (clone 73B, mouse IgG1, (Mackay, Marston & Dudler 1990) ) or the appropriate isotype control (mouse IgG1).The plate was then incubated at 4°C for 30 minutes and then washed as described previously and spun at 2000rpm for 1 minute. The cells were re-suspended in 100ul of secondary antibody (goat anti-mouse IgG-Alexa 647 [H+L], Invitrogen, Carlsbad, CA or rat anti-mouse IgG2a-PE). The plate was then incubated for 30 minutes at 4°C and then washed as described previously. Cells were re-suspended in 200ul of 10% normal rat serum in FACS buffer and incubated at 4°C for 30 minutes. After spinning the cells were re-suspended in 100ul of either anti-ovine CD4 mAb conjugated to FITC (clone 44.38, mouse IgG2a, AbDserotec) or the mouse IgG2a-FITC isotype control. The plate was incubated at 4°C for 30 minutes before washing as described previously and cells were re- suspended in 200ul PBS solution and spun again at 2000rpm for 1 minute. Cells were fixed by adding 200ul 1% PFA in PBS and incubating the plate at room temperature for 10 minutes before spinning at 2000rpm for 1 minute and re-suspension in 200ul PBS solution. . At this point the plate was covered and stored at 4°C until being read on a BD FACSCanto II® flowcytometer (BD Biosciences, San Jose, California, USA) within 48 hours.

For the Treg (CD4+CD45RA+Foxp3) stain cells were re-suspended and incubated at 4°C for 30 minutes with 100ul of either anti-ovine CD4 mAb conjugated to FITC (mouse IgG2a), anti-ovine mAb to CD45RA (clone 73B, mouse IgG1, (Mackay, Marston & Dudler 1990) ) or the appropriate isotype control (mouse IgG1 o mouse IgG2a-FITC). The plate was then incubated at 4°C for 30 minutes and washed as described previously. Cells were re-suspended in 100ul of the secondary antibody (Goat anti-mouse IgG1-PE). The plate was incubated for 30 minutes at 4°C and washed as described previously before cells were re-suspended in 200ul PBS solutions and spun at 2000rpm for 1 minute. Cells were fixed by adding 200ul 1% PFA in PBS and incubating the plate at room temperature for 10 minutes before spinning at 2000rpm for 1 minute and re-suspension in 200ul PBS solution. After spinning at 2000rpm for 1 minute cells were re-suspended in 200ul permeabilisation solution (PBS + 0.2% saponin + 20% NRS) and incubated 4°C for 14-18 hours and spun at 2000rpm for 1 minute before resuspension goat anti-rat Foxp3 mAb conjugated to Alexaflour647 (goat anti-rat IgG2a-647). The plate then incubated for 1 hour at 4°C before washing as described previously. Cells were re-suspended in 200ul PBS solutions and spun at 2000rpm for 1 minute. Cells were fixed by adding 200ul 1% PFA in PBS and incubating the plate at room temperature for 10 minutes before spinning at 2000rpm for 1 minute and re-suspension in 200ul PBS solution. At this point the plate is covered and stored at 4°C until being read on a BD FACSCanto II® flowcytometer (BD Biosciences, San Jose, California, USA) within 48 hours.

Our single stain assays were performed within two months of returning from the field in late August 2011, but due to time taken optimising our multi-stain assays these were run seven months after fixation. Both sets of assays produce estimates of the proportion of CD4+ and CD8+, and to check that samples had not degraded by the time we ran our multi-stain assays we examined the correlation between these proportions. These were reasonably high, suggesting no meaningful degradation of the fixed samples had occurred: r = 0.56 for CD4+ and r = 0.71 for CD8+).

**Supplementary Table 1.** Summary of total individuals sampled and numbers of samples passing quality control for each of the 11 immune parameters measured in this study.

| **Immune**  **Marker** | **Total number sampled** | **Number of samples passing quality control** | | |
| --- | --- | --- | --- | --- |
|  |  | **Male** | **Female** | **Total** |
| **Neutrophil:Lymphocyte** | 282 | 76 | 145 | 221 |
| **Eosinophil** | 282 | 76 | 146 | 222 |
| **CD4+** | 200 | 49 | 139 | 188 |
| **CD8+** | 200 | 48 | 129 | 177 |
| **CD4+ naïve** | 200 | 49 | 130 | 179 |
| **CD8+ naïve** | 200 | 49 | 130 | 179 |
| **γδ+ TcR** | 200 | 47 | 130 | 177 |
| **Treg** | 200 | 49 | 139 | 188 |
| **Anti-Tc IgA** | 287 | 94 | 187 | 281 |
| **Anti-Tc IgE** | 287 | 96 | 187 | 283 |
| **Anti-Tc IgG** | 287 | 96 | 185 | 281 |

**Supplementary Table 2.** Principal component analysis of the 11 immune parameters measured in this study. The standard deviation and proportion of variance explained by each axis is shown in the upper table, and the loadings of each variable on each axis are displayed in the lower part of the table.

|  | PC1 | PC2 | PC3 | PC4 | PC5 | PC6 | PC7 | PC8 | PC9 | PC10 | PC11 |
| --- | --- | --- | --- | --- | --- | --- | --- | --- | --- | --- | --- |
| Standard deviation | 1.962 | 1.1572 | 1.065 | 0.983 | 0.908 | 0.893 | 0.850 | 1.743 | 0.622 | 0.560 | 0.335 |
| Proportion of variance | 0.350 | 0.122 | 0.103 | 0.088 | 0.075 | 0.073 | 0.066 | 0.050 | 0.035 | 0.028 | 0.010 |
| Cumulative proportion of variance | 0.350 | 0.472 | 0.575 | 0.663 | 0.738 | 0.810 | 0.846 | 0.926 | 0.961 | 0.990 | 1.000 |
|  | **Loadings** | | | | | | | | | | |
| Immune Marker | **PC1** | **PC2** | **PC3** | **PC4** | **PC5** | **PC6** | **PC7** | **PC8** | **PC9** | **PC10** | **PC11** |
| γδ+ TcR | 0.393 | -0.118 | 0.182 | -0.013 | -0.080 | 0.178 | -0.356 | -0.014 | -0.706 | 0.316 | -0.187 |
| CD4+ | -0.145 | 0.329 | -0.287 | -0.684 | -0.420 | 0.169 | 0.219 | 0.082 | -0.223 | -0.061 | -0.083 |
| CD4+naïve | 0.447 | 0.041 | 0.088 | -0.128 | -0.297 | 0.030 | -0.095 | -0.309 | 0.152 | -0.027 | 0.746 |
| CD8+ | -0.232 | -0.371 | 0.206 | -0.432 | -0.206 | -0.479 | -0.931 | -0.105 | 0.247 | 0.255 | -0.136 |
| CD8+ naïve | 0.415 | 0.228 | 0.142 | 0.046 | 0.218 | 0.070 | 0.037 | -0.426 | 0.352 | -0.155 | -0.608 |
| Treg | 0.061 | -0.648 | 0.007 | 0.118 | -0.461 | 0.412 | 0.221 | 0.306 | 0.171 | -0.035 | -0.065 |
| Neutrophil:Lymphocyte | -0.175 | 0.071 | -0.680 | 0.301 | -0.306 | 0.034 | -0.298 | -0.237 | 0.061 | 0.336 | -0.013 |
| Eosinophil | -0.282 | -0.326 | -0.067 | -0.222 | 0.335 | 0.413 | -0.100 | -0.599 | -0.66 | -0.204 | 0.016 |
| Anti-Tc IgA | -0.314 | 0.236 | 0.275 | 0.178 | -0.294 | 0.179 | -0.566 | 0.176 | -0.026 | -0.256 | 0.036 |
| Anti-Tc IgE | -0.306 | -0.075 | 0.250 | 0.290 | -0.365 | -0.321 | 0.425 | -0.405 | -0.400 | -0.100 | 0.040 |
| Anti-Tc IgG | -0.305 | 0.309 | 0.456 | 0.047 | -0.037 | 0.407 | 0.109 | -0.033 | 0.204 | 0.612 | 0.070 |

**Figure S1.** An example of the flow cytometry gating strategy and staining patterns used in this study. **A:** FACS density plot of cell counts sorted by forward (FSC) and side (SSC) scatter, with the gate used to isolate the lymphocyte sub-population from the granulocytes (directly above gate) and presumed debris (to the left of the gate). **B-D:** Examples of staining patterns within the lymphocyte gate with different fluorophore-labelled antibodies used to identify lymphocyte sub-populations. **B:**  The gating of the CD8 T cells (labelled with PE) against CD4 T cells (FITC), used to calculate the proportion of each type of cell; **C:** The gating to identify CD45RA (APC label) positive and negative CD4 T cells; **D:** Having excluded all non-CD8 positive cells (gates in panel B), this plot shows an example of the separation of CD45RA positive and negative CD8 T cells.
